# The 1450-nm Diode Laser Reduces Redness and Porphyrin Density: An Image-Based, Patient-Oriented Appraisal

**DOI:** 10.3390/jcm12134500

**Published:** 2023-07-05

**Authors:** Gong-Yau Chu, Chieh-Chen Huang, Nai-Hua Shih, Chung-Hua Hsu, Ching-Ying Wu

**Affiliations:** 1 Institute of Traditional Medicine, School of Medicine, National Yang-Ming Chiao Tung University, Taipei 30010, Taiwan; aguest122@hotmail.com.tw; 2 Institute of Traditional Medicine, School of Medicine, National Yang-Ming University, Taipei 30010, Taiwan; 3 Department of Dermatology, Shin-Kong Wu Ho-Su Memorial Hospital, Taipei 11101, Taiwan; 4 Department of Dermatology, Kang-Ning General Hospital, Taipei 114, Taiwan; 5 Department of Laboratory Medicine, Kaohsiung Municipal Ta-Tung Hospital, Kaohsiung 80145, Taiwan; 6 Department of Chinese Medicine, Taipei City Hospital, Linsen Chinese Medicine and Kunming Branch, Taipei 104, Taiwan; 7 Department of Dermatology, Kaohsiung Municipal Ta-Tung Hospital, Kaohsiung Medical University Hospital, Kaohsiung Medical University, Kaohsiung 80756, Taiwan; 8 Department of Dermatology, College of Medicine, Kaohsiung Medical University, Kaohsiung 80756, Taiwan; 9 Department of Cosmetic Science, Chang Gung University of Science and Technology, Taoyuan 61363, Taiwan

**Keywords:** acne vulgaris, inflammatory acne, 1450-nm diode laser

## Abstract

Background: Acne vulgaris remains the leading dermatological condition. The efficacy of laser treatment has been supported by many clinical studies, but studies investigating its multidimensional action are lacking. Aim: To comprehensively investigate the efficacy of 1450-nm diode laser treatment in patients with inflammatory acne and provide objective and subjective data for doctors in clinical practice. Methods: This retrospective study included patients with inflammatory acne lesions who underwent three courses of 1450-nm diode laser treatment between October 2019 and August 2020. Facial surface analysis was performed via objective computer assessments using the Canfield VISIA imaging system. Post-treatment subjective assessments were retrieved and analyzed using the clinical global impression–improvement index (CGI-I) and patient global impression of improvement scales (PGI-I). Results: The final analysis included 20 patients. The changes in the porphyrin VISIA system scores demonstrated significant improvement, with median scores being 35.83, 48.83, and 54.83, respectively. The changes in the red area VISIA scores also showed improvement, with the median scores being 48, 50.33, and 58.83, respectively. The average CGI-I scale scores were 2.2 ± 1.01, 1.70 ± 0.80, and 1.50 ± 0.76, respectively (*p* = 0.001), and the average PGI-I scale scores were 3.10 ± 0.85, 3.10 ± 0.97, and 3.05 ± 0.95, respectively (*p* = 0.727), with no significant changes observed in sebum production. Conclusions: The present study is the first to provide objective and subjective evidence proving that the 1450-nm diode laser can reduce inflammatory acne lesions.

## 1. Introduction

Acne vulgaris remains the leading clinical dermatological outpatient condition [1], significantly affecting both the quality of life and self-esteem of teenagers. Acne, or acne scarring, may persist till adulthood, resulting in a long-term impact. The underlying mechanism of acne includes multiple etiologies involving the pilosebaceous unit. The four main scenarios include increased sebum production, the hyperkeratinization of the hair follicle, the follicular accumulation of *Cutibacterium acnes* (*C. acnes*), and inflammatory reaction induced by multiple cytokines [2].

The common topical acne therapies in clinical practice include antibiotics, benzoyl peroxide, salicylic acid, azelaic acid, retinoids, and sulfone agents. Owing to the complexity and persistence of acne, single therapy is usually insufficient for achieving satisfactory efficacy. Systemic antibiotics have been used most commonly in acne therapy for years and are typically used, often in combination with a topical retinoid and benzoyl peroxide, in patients with moderate to severe inflammatory acne. Antibiotics that are effective against acne include doxycycline, minocycline, tetracycline, trimethoprim/sulfamethoxazole, trimethoprim, azithromycin, erythromycin, cephalexin, and amoxicillin [3]. The tetracycline class of antibiotics is regarded as first-line treatment in moderate to severe acne except contraindications (i.e., allergy, ≤8 years of age, or pregnancy). These antibiotics have the ability to inhibit protein synthesis by binding the 30S subunit of the bacterial ribosome and have anti-inflammatory effects in acne lesions. The structure of trimethoprim is similar to folic acid, which can interfere with the enzyme dihydrofolate reductase. Sulfamethoxazole is able to block the bacterial synthesis of folic acid. These two compounds have synergistic effects on blocking nucleotide and amino acid synthesis in the bacteria. Erythromycin and azithromycin are also effective at treating acne. The anti-acne mechanism for the macrolide class of antibiotics binds the 50S subunit of the bacterial ribosome. In clinical practice, penicillins and cephalosporins are sometimes used as alternative anti-acne treatments. The mechanism of action for these antibiotics is binding the penicillin-binding proteins in the bacterial cell membrane and blocking bacterial cell wall synthesis [3]. However, some patients experience side effects and resistance to systemic antibiotics.

Combination oral contraceptive pills (COCs) are currently used to treat patients with acne. The mechanism of COCs in the treatment of acne is based on their antiandrogenic properties, but their use also results in an increased risk of cardiovascular events and breast cancer, which cannot be neglected [3]. Many countries have approved isotretinoin for the treatment of severe recalcitrant acne. Oral isotretinoin is an isomer of retinoic acid and has proven to have positive efficacy for most patients with moderate to severe acne. It is also able to successfully treat recalcitrant acne that is treatment-resistant to oral antibiotic therapy. This drug decreases sebum secretion, thereby reducing acne lesions and acne scarring. Its common side effects involve the mucocutaneous, musculoskeletal, and ophthalmic systems, and considering its well-known teratogenic effects, the drug is used only for severe recalcitrant acne [3].

Following the observation that sunlight may be beneficial in treating acne, dermatologists have treated acne lesions using light- or laser-based therapies [4]. The observed effects may be due to the alteration of the sebaceous gland structure, the destruction of bacterial colonization, or both. *C. acnes* is a gram positive microaerophilic skin bacterium and the main pathogenesis of acne. It can produce endogenous porphyrins, and these porphyrins generate reactive free radical species to cause bacterial devastation after absorbing light energy at specific wavelengths. These light sources include narrowband light devices, IPL devices (broadband light), KTP lasers (532 nm), PDLs (585–595 nm), and various orange/red light lasers or light sources (610–635 nm). Other light/laser therapies targeting the sebaceous gland to produce a reduction in size and sebum output can lead to acne improvement. The devices include infrared lasers, the 1450-nm diode laser, the 1540-nm erbium glass laser, intense pulsed light, radiofrequency, and photodynamic therapy. Laser- and light-based acne treatments are good options for non-responders to medical treatments and those patients who have side effects or contraindications for oral medications [4,5,6,7]. The use of the 1450-nm diode laser is one of the most promising treatments for acne. Its effect is based on the thermal coagulation of skin tissue in the mid-third of the dermis to a depth of 500 µm. This thermal effect results in the destruction of the sebaceous gland and the reduction of sebaceous gland activity, subsequently reducing inflammatory acne lesions [8]. Clinical experience shows that the use of the 1450-nm diode laser can reduce inflammatory acne lesions, and many clinical studies have corroborated the laser’s efficacy [9,10,11,12,13,14,15].

To explore the multidimensional outcomes of 1450-nm diode laser treatment, we analyzed patients with inflammatory acne lesions after 1450-nm diode laser treatment. The VISIA imaging system, clinician’s assessments, and patients’ self-evaluation were used for the assessment. The aim of this study was to comprehensively investigate the efficacy of the 1450-nm diode laser for patients with inflammatory acne and to provide both objective and subjective data for doctors in clinical practice.

## 2. Materials and Methods

### 2.1. Study Design

This study was conducted at the Kaohsiung Medical University Hospital and was approved by the institutional review board of Kaohsiung Medical University Hospital. Informed consent was obtained from all patients. All patients with inflammatory acne aged 20–50 years who completed three courses of full-face 1450-nm diode laser treatment between October 2019 and August 2020 with a treatment interval of 4–6 weeks were included. We also collected data including demographics, skin type, medical history, previous acne treatments, a 1450-nm diode laser protocol (Smoothbeam; Candela Corporation, Marlborough, MA, USA), a treatment protocol (with an integrated dynamic cooling device, repetition rate of 1 Hz, and a pulse duration of 210 milliseconds. Each patient received treatments with single pulses, delivered without overlap. The treatment fluence was 14 to 18 J/cm^2,^ and the spot size was 6 mm. The cryogen spray cooling device setting was set at 40 ms to cool the skin and reduce side effects, and patient photographs were taken before treatment and at follow-up visits. Patients who underwent any concurrent acne treatments or other laser or device procedures during the course of the 1450-nm diode laser treatment were excluded.

In clinical practice, facial images were taken before the patients’ next laser therapy to present the result of the last treatment. Facial surface analysis was performed as objective computer assessments using the Canfield VISIA with the Canfield imaging system (Canfield Scientific, Inc., Parsippany-Troy Hills, NJ, USA). We performed eight surface analyses (porphyrins, red areas, wrinkles, pores, texture, spots, ultraviolet (UV) spots, and brown spots) using color, UV, and cross-polarized photographs. We systematically obtained three types of photographs: standard flash, cross-polarized, and ultraviolet A (UVA) lighting. All of the patients received full-face treatment, and we divided the face into the right, middle, and left areas for further analysis at each time point and obtained an average value for that treatment.

Porphyrins are bacterial metabolites that can become embedded in pores and cause acne. They can be distinguished using UV light and can be helpful in selecting ideal treatments for acne. Red areas are caused by blood vessels that circulate in the papillary dermis, which might be related to various conditions such as inflammatory reactions. Wrinkles imply grooves in the skin associated with decreasing skin elasticity and aging. Pores are tiny ring-shaped surface openings in the skin. Texture reflects the smoothness of the skin. Spots include brownish or reddish skin lesions such as freckles, hyperpigmentation, and vascular lesions; they differ in size and shape and are usually visible. UV spots are not typically visible to the naked eye in normal light. These spots are the results of sun damage. Brown spots, including freckles, moles, and melasma, result from excess melanin deposition beneath the skin surface. The VISIA system measures spots, porphyrins, wrinkles, pores, texture, UV spots, red areas, and brown spots. Higher scores indicate a higher percentile measurement position from the bottom and represent improvement.

In the clinical setting, the doctor typically evaluates the degree of improvement in inflammatory acne lesions and records the patient’s self-assessment of oil secretion on the face (at approximately 4–6 weeks after the last treatment) before deciding on the next treatment step. Post-treatment subjective assessments were retrieved from the patient’s chart and subsequently analyzed. The results of the dermatologist’s evaluation were captured using the clinical global impression–improvement (CGI−I) scale (1–7 scale, wherein 1 = very much improved and 7 = very much worse; Table 1). Patient self-assessment responses were recorded based on the patient global impression of improvement (PGI−I) scale (1–7 scale, wherein 1 = very much improved and 7 = very much worse; Table 2).

### 2.2. Statistical Analysis

Since the number of subjects is less than 30, we use the nonparametric statistical tests. Data are presented as the median with an interquartile range (IQR) at each time point. Mann–Whitney tests were used to compare the difference in age between genders. The Wilcoxon signed rank test was used to compare the changes between the first and the second time point, the first and the third time point, and the second and the third time point, in spots, wrinkles, texture, pores, UV spots, brown spots, red areas, and porphyrins by VISIA scores. We also analyzed the results of the CGI−I and PGI−I scales using analysis of variance. *p*−values of <0.05 were considered significant. Statistical analysis was performed using SPSS version 19.0 (SPSS, Chicago, IL, USA).

## 3. Results

A total of 26 patients (11 men and 15 women) treated between October 2019 and August 2020 met the inclusion criteria. We excluded six patients because of incomplete records. In total, 20 patients were included in the final analysis (7 men and 13 women). Table 3 presents their demographics. Their mean age was 31.05 ± 9.80 years. No significant difference was found between men and women according to the results of the Mann–Whitney test (median age: 32.0 vs. 28.0, *p* = 0.284). Fitzpatrick skin types III (n = 12) and IV (n = 8) were observed. The mean laser frequency was 16.38 J/cm^2^ (Table 3).

### 3.1. Results of the VISIA System Analysis

**Figure 1:**
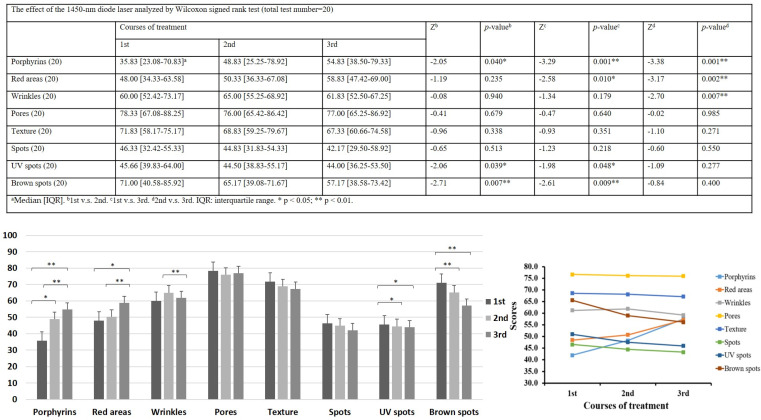
Effect of the 1450-nm diode laser analyzed by Wilcoxon signed rank test. VISIA system scores of the changes in porphyrin levels, red areas, wrinkles, pores, texture, spots, UV spots, and brown spots. (1st: after the 1st laser treatment for 4−6weeks and before the 2nd laser treatment; 2nd: after the 2nd laser treatment for 4−6weeks and before the 3rd laser treatment; and 3rd: after the 3rd laser treatment for 4−6 weeks) * *p* < 0.05; ** *p* < 0.01.

The changes in the porphyrin VISIA system scores demonstrated considerable improvement at each time point; the median scores were 35.83, 48.83, and 54.83, respectively. Furthermore, the changes in red area VISIA scores showed remarkable improvement, with median scores of 48, 50.33, and 58.83, respectively. The VISIA wrinkle scores seemed to display statistical significance in comparison with the second and the third time point, and the median scores were 60, 65, and 61.83, respectively. No significant changes were noted in pores, texture, and spots. Nevertheless, the changes in UV spot VISIA scores demonstrated significant deterioration in comparison with the first and the second time point and the first and the third time point, and the median scores were 45.66, 44.50, and 44. The brown spot VISIA scores also showed considerable deterioration in comparison with the first and the second time point and the first and the third time point, with median scores of 71, 65.17, and 57.17, respectively (Figure 1).

### 3.2. CGI−I Scale Result

**Figure 2:**
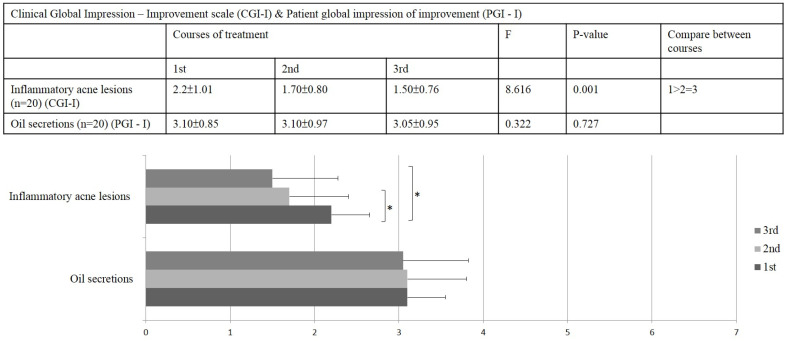
Results of the clinical global impression–improvement (CGI−I) scale and the patient global impression of improvement (PGI−I) scale (1–7 scale, wherein 1 = very much improved and 7 = very much worse) * *p* < 0.05.

The results of the CGI−I scale indicated that, based on the dermatologist’s evaluation, most patients demonstrated a significant improvement in inflammatory acne lesions after the 1450-nm diode laser treatment. The average scores were 2.2 ± 1.01, 1.70 ± 0.80, and 1.50 ± 0.76, respectively (*p* = 0.001; Figure 2).

### 3.3. PGI-I Improvement Result

The results of the PGI−I scale showed that most patients reported only little improvement in oil secretion on the face, and no significant changes were found with any of the 1450-nm diode laser treatments. The average scores were 3.10 ± 0.85, 3.10 ± 0.97, and 3.05 ± 0.95, respectively (*p* = 0.727; Figure 2).

## 4. Discussion

In this study, we examined the multidimensional aspects of 1450-nm diode laser treatment for inflammatory acne via the objective VISIA image system and subjective patient-oriented evaluation. To the best of our knowledge, this is the first combined research study to provide a comprehensive evaluation of the 1450-nm diode laser for acne treatment.

The 1450-nm diode laser is commonly used to treat patients with inflammatory acne lesions [9,10,11,12,13,14,15]. The heating and breaking of the sebaceous gland and associated structures, causing a reduction in the sebaceous gland activity and a subsequent decrease in inflammatory acne lesions, is the presumed key mechanism of acne improvement [4]. However, the mechanism by which the 1450-nm diode laser works to reduce inflammatory acne lesions remains ambiguous. Paithankar et al. reported that a histopathological examination 1 and 3 days after treatment revealed destruction in the dermis and thermal changes in the sebaceous glands, but the epidermis remained intact. However, the histopathological examination of biopsies obtained after 7 days demonstrated that the sebaceous glands recover from the initial injury to achieve an intact appearance. In this study, whether the destruction of the sebaceous glands was the major effect of the 1450-nm diode laser in controlling acne is questionable [16]. In a split-face study, three 1450-nm laser treatment sessions did not result in substantial changes in the rate of facial sebum excretion, but the laser sessions reduced sebum production on both the laser-treated and control sides at 1 month post treatment [17]. In another study, an 18% reduction in sebum output was observed after three 1450-nm diode laser treatments. Therefore, it was concluded that direct destruction of the sebaceous glands on the laser-treated side was unlikely to be the main effect resulting in the control of acne lesions; thus, other mechanisms should be considered to describe the clinical efficacy of this treatment for acne vulgaris [17]. The 1450-nm diode laser might have other modes of action in the treatment of acne vulgaris [18].

**Figure 3:**
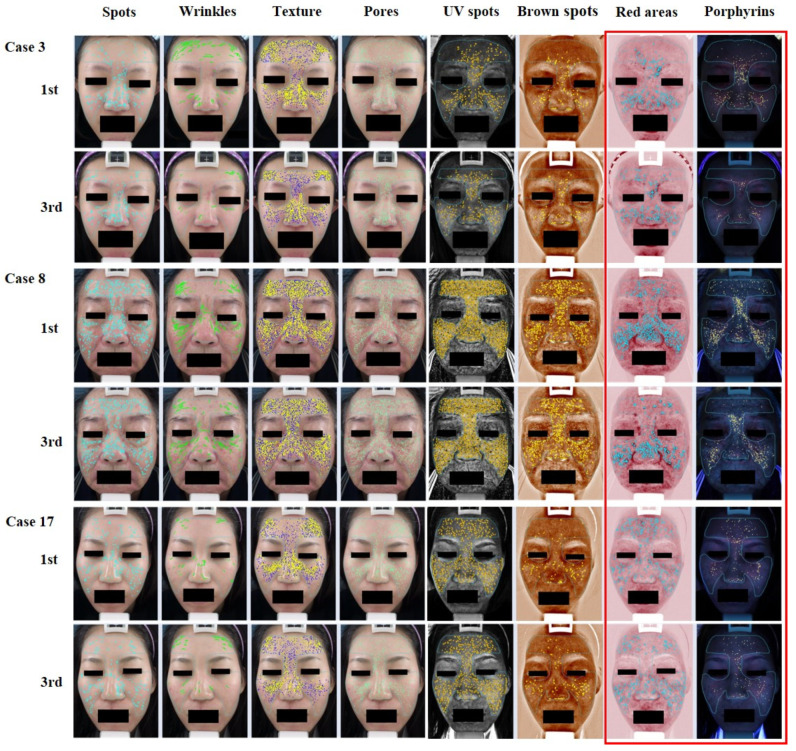
VISIA images. The images show substantial improvement in porphyrin levels and red areas.

The original theory remains unconfirmed, and studies discussing the possible antiacne mechanism of the 1450-nm diode laser are lacking. The VISIA system scores in our study revealed significant differences in the reduction in red areas and porphyrin density (Figure 1 and Figure 3). The results of the physician’s evaluation also revealed a significant improvement in acne lesions after the 1450-nm diode laser treatment (Figure 2). However, the patients did not record a significant difference in sebum production (Figure 2). Our results corroborate those of previous clinical studies, suggesting that the destruction of the sebaceous glands and the reduction in oil production might be not the key mechanisms of action of the 1450-nm diode laser in reducing inflammatory acne lesions [16,17,18]. The VISIA system analysis also revealed an interesting result: that porphyrin levels decrease significantly after the 1450-nm diode laser treatment (Figure 1 and Figure 3). Because porphyrin is produced by *C. acnes*, its density is associated with the pilosebaceous follicles and strongly reveals the population density of *C. acnes* in acne lesions [5]. *C. acnes* is an important contributor among the microbiota of the pilosebaceous unit, with higher numbers of *C. acnes* observed in patients with acne than in healthy individuals. The observed dysbiosis in the sebaceous follicles has been suggested to lead to the development of acne lesions [19]. To promote its growth, *C. acnes* uses the secreted sebum as a metabolic substrate. It further enhances sebum secretion and breaks down triglycerides that are secreted from the sebaceous glands [20]. *C. acnes* biofilms have been observed in acne, particularly after long-term antibiotic treatment. Biofilms offer protection from immune system attack and are more advantageous than free-living bacteria for survival in harsh environments because they can facilitate intercellular communications, enhance proinflammatory properties, increase adhesion to the follicular wall, resist antibiotics, and more easily obtain nutrients [19]. Therefore, the eradication of *C. acnes* biofilms can greatly reduce inflammatory acne lesions and may serve as a critical mechanism for antiacne treatment in the future [21,22,23,24].

Our study demonstrated a significant improvement in inflammatory acne lesions with the decrease of porphyrin density in patients after 1450-nm diode laser treatment. This finding has not been discussed in the literature. According to the limited existing evidence, we speculate that the 1450-nm diode laser may reduce bacterial growth. The generation of reactive free radical species, subsequently causing bacterial damage, is unlikely to be the underlying mechanism because they are not absorbed by porphyrin at this wavelength [4]. However, the ability of the 1450-nm diode laser to destroy the sebaceous glands is proved by histological examination [16]. We hypothesize that the destruction of the sebaceous glands may simultaneously interfere with the growth of *C. acnes*. Another possible mechanism is the inhibition of biofilm formation associated with the damage of the sebaceous glands. The breakdown of the biofilm leading to reduced protection may facilitate *C. acnes* eradication. After *C. acnes* interference is decreased, the inflammatory reaction subsequently subsides and sebaceous gland activity recovers to the original state. Hence, our results showed the 1450-nm diode laser can result in significant improvement in inflammatory acne lesions but only a minor improvement in the oily nature of the skin. The definite mechanisms are still poorly understood, and further investigations are warranted.

Although the 1450-nm diode laser cannot influence melanin formation, more UV spots and brown spots were observed following treatment by VISIA scores analysis. This might be related to the fact that it is easy to detect those spots due to a decrease in acne lesions and red areas. Patients might be willing go to outside frequently because of the improvement in acne lesions. The wrinkles VISIA scores seemed to be a conflict after 1450-nm diode laser treatment. Thus, we did not think the data had clinical significance. Hence, we cannot conclude whether the deterioration in UV spots, brown spots, and wrinkles was due to the treatment. Further, no significant differences were present in other evaluation indexes, including pores, textures, and spots, in patients after 1450-nm diode laser treatment (Figure 1). The limitations of the study include the small sample size and the open-label study design. However, the research team still noticed a clinical significance in the study. Future large-scale, double-blind, randomized controlled trials are warranted to better understand the efficacy of the 1450-nm diode laser in inflammatory acne lesions.

## 5. Conclusions

In this study, we found a significant reduction in red acne lesions and a decrease in porphyrin density in patients after 1450-nm diode laser treatment. According to clinical assessment and patient self-evaluation, considerable clinical improvement was observed in the inflammatory acne lesions without perceptible sebum reduction. This is the first study to provide comprehensive evidence that the 1450-nm diode laser can reduce inflammatory acne lesions. Further, there appears to be an interesting and potential correlation between 1450-nm diode laser treatment and a reduction of *C. acne* numbers, suggesting that patients with inflammatory acne can benefit from this treatment.

## Figures and Tables

**Table 1:** Clinical global impression−improvement scale.

1	Very much better
2	Much better
3	A little better
4	No change
5	A little worse
6	Much worse
7	Very much worse

**Table 2:** Patient global impression of improvement scale.

1	Very much better
2	Much better
3	A little better
4	No change
5	A little worse
6	Much worse
7	Very much worse

**Table 3:** Demographic and clinical characteristics of patients receiving 1450-nm diode laser.

Characteristic	Female, n = 13	Male, n = 7	*p*-Value
Age, mean ± standard deviation	29.46 ± 9.94	34.00 ± 9.56	0.337
Age, Median (IQR)	28.0 (23−37)	32.0 (24−43)	0.284 ^a^
Fitzpatrick skin type, n (%)			
I	0	0	
II	0	0	
III	9 (69.23%)	3 (42.86%)	
IV	4 (30.77%)	4 (57.12%)	
V	0	0	
VI	0	0	
Mean laser fluency, J/cm^2^			
Treatment 1	16.0	16.29	
Treatment 2	16.23	16.5	
Treatment 3	16.38	16.86	

^a^ Mann–Whitney test. IQR: interquartile range.

## Data Availability

The authors confirm that the data supporting the findings of this study are available within the article.

## References

[1] Koo J.Y., Smith L.L. (1991). Psychological aspects of acne. Pediatr. Dermatol..

[2] Thiboutot D., Gollnick H., Bettoli V., Dréno B., Kang S., Leyden J.J., Shalita A.R., Lozada V.T., Berson D., Finlay A. (2009). New insights into the management of acne: An update from the global alliance to improve outcomes in acne group. J. Am. Acad. Dermatol..

[3] Zaenglein A.L., Pathy A.L., Schlosser B.J., Alikhan A., Baldwin H.E., Berson D.S., Bowe W.P., Graber E.M., Harper J.C., Kang S. (2016). Guidelines of care for the management of acne vulgaris. J. Am. Acad. Dermatol..

[4] Rai R., Natarajan K. (2013). Laser and light based treatments of acne. Indian. J. Dermatol. Venereol. Leprol..

[5] Piérard-Franchimont C., Paquet P., Piérard G.E. (2011). New approaches in light/laser therapies and photodynamic treatment of acne. Expert Opin. Pharmacother..

[6] Elman M., Lebzelter J. (2004). Light therapy in the treatment of acne vulgaris. Dermatol. Surg..

[7] Ross E.V. (2005). Optical treatments for acne. Dermatol. Ther..

[8] Jih M.H., Kimyai-Asadi A. (2007). Laser treatment of acne vulgaris. Semin. Plast. Surg..

[9] Friedman P.M., Jih M.H., Kimyai-Asadi A., Goldberg L.H. (2004). Treatment of inflammatory facial acne vulgaris with the 1450-nm diode laser: A pilot study. Dermatol. Surg..

[10] Jih M.H., Friedman P.M., Goldberg L.H., Robles M., Glaich A.S., Kimyai-Asadi A. (2006). The 1450-nm diode laser for facial inflammatory acne vulgaris: Dose–response and 12-month follow-up study. J. Am. Acad. Dermatol..

[11] Uebelhoer N.S., Bogle M.A., Dover J.S., Arndt K.A., Rohrer T.E. (2007). Comparison of stacked pulses versus double-pass treatments of facial acne with a 450-nm laser. Dermatol. Surg..

[12] Bernstein E.F. (2007). A pilot investigation comparing low-energy, double pass 1450-nm laser treatment of acne to conventional single-pass, high-energy treatment. Lasers Surg. Med..

[13] Konishi N., Endo H., Oiso N., Kawara S., Kawada A. (2007). Acne phototherapywith a 1450-nm diode laser: An open study. Ther. Clin. Risk Manag..

[14] Noborio R., Nishida E., Morita A. (2009). Clinical effect of low-energy double-pass 1450-nm laser treatment for acne in Asians. Photodermatol. Photoimmunol. Photomed..

[15] Kwon H.H., Park H.Y., Choi S.C., Bae Y., Jung J.Y., Park G.H. (2018). Novel device-based acne treatments: Comparison of a 1450-nm diode laser and microneedling radiofrequency on mild-to-moderate acne vulgaris and seborrhoea in Korean patients through a 20-week prospective, randomized, split-face study. J. Eur. Acad. Dermatol. Venereol..

[16] Paithankar D.Y., Ross E.V., Saleh B.A., Blair M.A., Graham B.S. (2002). Acne treatment with a 1,450-nm wavelength laser and cryogen spray cooling. Lasers Surg. Med..

[17] Laubach H.J., Astner S., Watanabe K., Clifford J., Rius-Diaz F., Zurakowski D., Manstein D. (2009). Effects of a 1,450-Nm diode laser on facial sebum excretion. Lasers Surg. Med..

[18] Perez-Maldonado A., Rünger T.M., Krejci-Papa N. (2007). The 1,450-nm diode laser reduces sebum production in facial skin: A possible mode of action of its effectiveness for the treatment of acne vulgaris. Lasers Surg. Med..

[19] Aslan Kayiran M.A., Karadag A.S., Al-Khuzaei S., Chen W.C., Parish L.C. (2020). Antibiotic resistance in acne: Mechanisms, complications and management. Am. J. Clin. Dermatol..

[20] Xu H., Li H. (2019). Acne, the skin microbiome, and antibiotic treatment. Am. J. Clin. Dermatol..

[21] Bernhardt M.J., Myntti M.F. (2016). Topical treatment with an agent disruptive to P acnes biofilmprovides positive therapeutic response: Results of a randomized clinical trial. J. Drugs Dermatol..

[22] Hazarika N. (2021). Acne vulgaris: New evidence in pathogenesis and future modalities of treatment. J. Dermatolog Treat..

[23] Fernandes C., Macedo D., Assis L. (2021). Antimicrobial photodynamic therapy against Propionibacterium acnes biofilms using hypericin (hypericum perforatum) photosensitizer: In vitro study. Lasers Surg. Med..

[24] Feuillolay C., Pecastaings S., Le Gac C., Fiorini-Puybaret C., Luc J., Joulia P., Roques C. (2016). A Myrtus communis extract enriched in myrtucummulones and ursolic acid reduces resistance of Propionibacterium acnes biofilms to antibiotics used in acne vulgaris. Phytomedicine.

